# Drug repositioning and ovarian cancer, a study based on Mendelian randomisation analysis

**DOI:** 10.3389/fonc.2024.1376515

**Published:** 2024-04-08

**Authors:** Lin Zhu, Hairong Zhang, Xiaoyu Zhang, Ruoqing Chen, Lei Xia

**Affiliations:** ^1^ School of Chinese Medicine, Shandong University of Traditional Chinese Medicine, Jinan, China; ^2^ Department of Obstetrics and Gynecology, Shandong Provincial Third Hospital, Jinan, China; ^3^ Department of Pathology, Shandong University of Traditional Chinese Medicine, Jinan, China

**Keywords:** Mendelian randomisation (MR) analysis, ovarian cancer, drug repositioning, statins, aspirin

## Abstract

**Background:**

The role of drug repositioning in the treatment of ovarian cancer has received increasing attention. Although promising results have been achieved, there are also major controversies.

**Methods:**

In this study, we conducted a drug-target Mendelian randomisation (MR) analysis to systematically investigate the reported effects and relevance of traditional drugs in the treatment of ovarian cancer. The inverse-variance weighted (IVW) method was used in the main analysis to estimate the causal effect. Several MR methods were used simultaneously to test the robustness of the results.

**Results:**

By screening 31 drugs with 110 targets, FNTA, HSPA5, NEU1, CCND1, CASP1, CASP3 were negatively correlated with ovarian cancer, and HMGCR, PLA2G4A, ITGAL, PTGS1, FNTB were positively correlated with ovarian cancer.

**Conclusion:**

Statins (HMGCR blockers), lonafarnib (farnesyltransferase inhibitors), the anti-inflammatory drug aspirin, and the anti-malarial drug adiponectin all have potential therapeutic roles in ovarian cancer treatment.

## Highlights

- The treatment outcomes for ovarian cancer remain unsatisfactory.
- Drug repositioning is a superior therapeutic strategy to investigate.
- Fat-soluble statins may be more effective than water-soluble statins.
- Statins (HMGCR blockers), lonafarnib (farnesyltransferase inhibitors), the anti-inflammatory drug aspirin, and the anti-malarial drug adiponectin all have a therapeutic role in ovarian cancer.

## Introduction

Ovarian cancer is the second most common gynaecological cancer and the gynaecological malignancy with the highest mortality rate worldwide (1). Due to the concealed location of the disease, most cases are found at an advanced stage. Although the effect of surgery or chemotherapy is better in early stage patients, they are prone to relapse and drug resistance. Therefore, there is a need to develop new therapeutic agents (2). But drug discovery and development is an expensive, time-consuming, and risky business. In this context, drug repositioning strategies are becoming increasingly attractive. Drug repositioning aims to identify new indications for old drugs, with the advantage that data on pharmacokinetic properties and toxicity are already available. Thus, in principle, it is possible to reduce research costs and accelerate drug use/distribution (3).

Currently, there are many related reports on the role of drug repositioning in ovarian cancer treatment (4, 5), such as lipid-lowering drug statins (6), glucose-lowering drug metformin (7), antiarrhythmic drug amiodarone (8), neuroprotective drugs (9), ACEI for hypertension (10), disulfiram for chronic alcoholism anti-inflammatory (11, 12), anti-bacterial (13, 14), anti-viral (15), and anti-parasitic drugs (16, 17) as well as calcium-channel blockers (18), have all shown different anti-ovarian cancer effects. However, there were huge differences in the specific results. This discrepancy may be related to being affected by different confounding factors (19).

Mendelian randomization studies are an etiological research method based on Mendel’s law of independent assignment, using genetic factors as instrumental variables for risk factors to make causal inferences about disease or health-related outcomes. Its advantage is that it can effectively avoid the influence of various confounding factors (20). In this study, we used the Mendelian randomization study method to analyse the previously reported therapeutic effects of conventional drugs on ovarian cancer.

## Materials and methods

**Figure 1:**
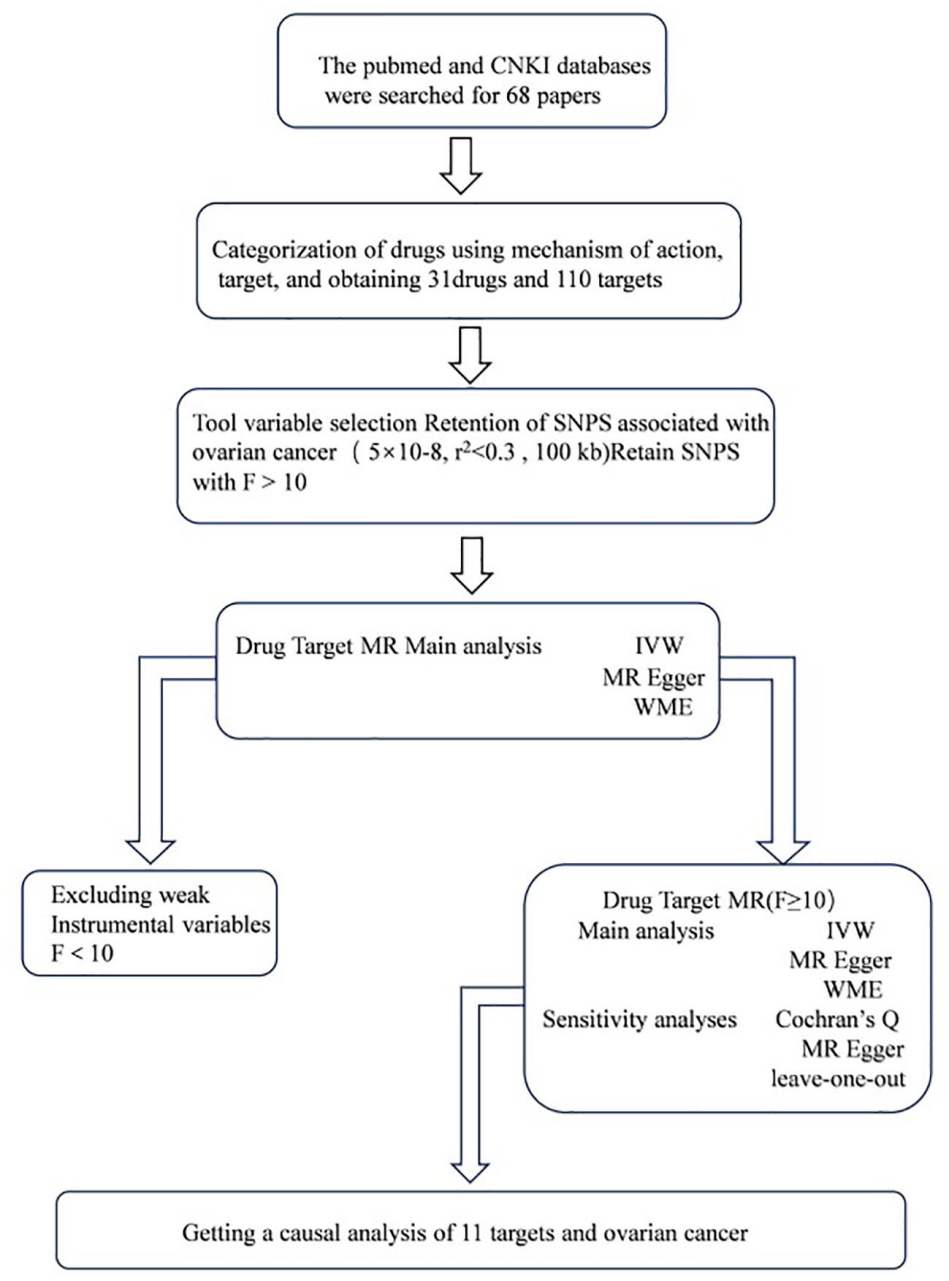
Work flows of the MR analysis for drug repositioning and ovarian cancer.

All analyses used publicly available database resources and therefore did not require ethical approval from an institutional review board. The entire MR analysis process illustrated in **Figure 1**.

### GWAS data source of ovarian cancer

Genetic association data for ovarian cancer (ieu-a-1120) were obtained from the Ovarian Cancer Association Consortium (OCAC), which performed a GWAS analysis of 22,406 cases of invasive epithelial ovarian cancer pathology and 40,941 healthy population controls, and through genetic analysis identified 17273705 SNP loci (21) (**Table 1**).

**Table 1:** Ovarian Cancer GWAS Data Sources and Descriptions from Open GWAS.

Outcome	Number of SNPs	Sample size	Sex	Population	Year	Data source	ID
**OC**	**17273705**	**63 347**	**Females**	**European**	**2017**	http://gwas.mrcieu.ac.uk/	**ieu-a-1120**
**OC**	**9822229**	**199741**	**Males and Females**	**European**	**2021**	**https://gwas.mrcieu.ac.uk/**	**ieu-b-4963**

We additionally selected a dataset on ovarian cancer (ieu-b-4963) from the UKB database, which contains 1,218 ovarian cancer cases and 198,523 controls from healthy populations, and identified 9,822,229 SNP loci by genetic analysis. Both data sets were from European populations. (**Table 1**) (https://gwas.mrcieu.ac.uk).

### Selection of drugs and targets

All publicly available literatures (published in English) were retrieved from China National Knowledge Infrastructure (CNKI) and Pubmed database from inception to December 2023 with the following keywords as search terms: “Drug repositioning” and “ovarian cancer”, and drugbank database was used to search the targets of related drugs. Drugs with the same mechanism of action and target were grouped into one group, and drugs with unclear targets were excluded.

### Selection of drug targeting sites and exposure factors

We used relevant drug targets as proxies for each drug exposure at 100 kb loci downstream of each of the drug target gene regions above and below the drug target gene region. p<5×10^-8^, r^2^<0.3 were obtained to screen for SNP loci closely related to the drug target as exposure factors, and targets for which no SNP loci were retrieved were excluded. To reduce the weak instrumental variable bias, the F-statistic was calculated separately for each SNP, and then weak instruments with an F-statistic < 10 were filtered.

### MR analysis

In univariate MR analysis, IVW is often used as the primary statistical method for MR analysis-. When horizontal pleiotropy is not present, inverse variance weighted (IVW) can provide relatively stable and accurate causality by using meta-analytical methods in combination with estimates of the causal effects of each SNP. The results of IVW were supplemented using the MR Egger, (weighted median, WME) method. Heterogeneity was estimated using Cochran’s Q statistic and p < 0.05 was used as the threshold parameter for identifying heterogeneity. Directional pleiotropy was detected using and MR-Egger test and outliers, and p < 0.05 was used as the threshold parameter for identifying horizontal pleiotropy. Leave-one-out sensitivity analyses were used to evaluate the magnitude of the effect of SNPs on the estimation results of causal associations.

## Result

After removing the drugs with completely consistent targets and those without clear targets, 31 drugs and 110 targets were obtained. (see **Supplementary Table 1**) The causal associations between different kinds of drug targets and ovarian cancer were analysed by MR, and there were 77 instrumental variable SNPs with F-statistics >10, suggesting that there was a low possibility of weak instrumental variable bias. The information of the 77 SNPs is shown in **Table 2**.

**Table 2:** Basic information on 77 relevant Instrumental variables.

TAGET	SNP	A1	β	sx	P	OC			R^2^	F
						β	sx	P		
**CASP1**	rs12792277	T	0.167217	0.0245762	1.02E-11	0.01975	0.02642	0.4549	0.003269087	12.89825794
	rs1792757	C	-0.120678	0.011986	7.64E-24	0.002906	0.01348	0.8293	0.007130507	28.24299502
	rs1977989	G	-0.117895	0.0133438	9.97E-19	0.0169	0.01501	0.2602	0.005499948	21.74885004
	rs34146979	A	0.628788	0.0207709	1.00E-200	-0.04773	0.02454	0.0518	0.060967617	255.3296149
	rs45447096	T	0.411869	0.0459424	3.11E-19	0.02542	0.0641	0.6917	0.005661645	22.39190328
	rs536909	G	0.220401	0.0157708	2.21E-44	-0.0367	0.01839	0.0459399	0.01364815	54.4157298
	rs61751523	C	-0.344218	0.0421618	3.24E-16	0.2393	0.07	0.000629796	0.004700033	18.57075032
**FNTA**	rs11992660	C	-0.215733	0.0371543	6.38E-09	0.03752	0.04683	0.423	0.002382856	16.68938072
	rs1648121	C	-0.126149	0.0177195	1.08E-12	0.05521	0.02031	0.00655707	0.003577908	25.08950211
	rs4623474	A	0.134011	0.0182798	2.28E-13	-0.05719	0.02104	0.00656296	0.003793203	26.60497444
	rs73629179	A	-0.209254	0.0376403	2.71E-08	0.02691	0.04675	0.564901	0.002184788	15.29908452
**HMGCR**	rs10059435	C	0.133038	0.0119377	7.64E-29	0.03744	0.01339	0.00517095	0.008722147	55.74625917
	rs111353455	A	0.169601	0.0203828	8.74E-17	0.02812	0.02323	0.2262	0.004881154	31.07672884
	rs4432861	A	0.193697	0.0253358	2.09E-14	0.03271	0.02957	0.2687	0.00412384	26.23518959
	rs6453133	G	0.190588	0.0127341	1.21E-50	0.01383	0.01448	0.3398	0.01562186	100.5445511
	rs72633962	C	0.188825	0.0169459	7.76E-29	0.000712	0.01968	0.9711	0.008719775	55.73097095
**PLA2G4A**	rs10798059	A	0.117349	0.0122064	7.00E-22	-0.005701	0.01476	0.699301	0.006505316	17.16480702
	rs12072973	G	0.217743	0.0117622	1.65E-76	0.02906	0.01374	0.03434	0.023703415	63.64513498
	rs12116694	T	-0.129099	0.0144351	3.77E-19	-0.01257	0.01759	0.4751	0.005634679	14.85454254
	rs12143166	G	-0.115354	0.0121911	3.02E-21	-0.01093	0.01433	0.4455	0.006303106	16.62787359
	rs2383556	C	-0.233405	0.0237386	8.17E-23	-0.02721	0.0263	0.3008	0.006802442	17.954168
	rs6678708	T	-0.168821	0.0153764	4.81E-28	-0.009744	0.0178	0.5841	0.008467731	22.38701942
**CASP3**	rs111227463	T	-0.401812	0.0179184	2.26E-111	0.000684242	0.000389656	0.0790005	0.034400213	49.03961383
	rs113437703	T	-0.282701	0.0176122	5.59E-58	0.000136613	0.000354897	0.7	0.017926209	25.12623486
	rs12512933	T	-0.537999	0.0162585	1.00E-200	0.000208812	0.000346837	0.55	0.07198997	106.7830683
	rs13110386	A	0.156414	0.0119188	2.42E-39	-0.000182119	0.00025253	0.47	0.012054263	16.79541169
	rs2171835	C	0.139911	0.0136906	1.62E-24	-0.000281797	0.000289852	0.33	0.007344767	0.18503819
	rs2696051	T	0.392627	0.0136244	1.28E-182	-0.000146342	0.000302736	0.630001	0.055567076	80.98964647
	rs4647626	A	-0.507488	0.0164636	1.00E-200	0.000507929	0.000353485	0.15	0.063070861	92.66272917
	rs4647673	A	-0.51248	0.0357271	1.15E-46	0.00144694	0.000791719	0.0680002	0.014367876	20.06599884
	rs72689292	T	-0.475223	0.0319518	4.92E-50	0.000569558	0.000739717	0.44	0.015430116	21.57276008
	rs72703555	C	-0.708763	0.0260002	1.26E-163	0.000109404	0.00051527	0.83	0.050013193	72.46863575
	rs755534	G	0.179249	0.0120407	3.99E-50	4.58E-05	0.000253844	0.86	0.015458467	21.6130201
**CCND1**	rs111929748	T	-0.25575	0.033154	1.22E-14	0.00197299	0.000565938	0.000490004	0.004198099	11.12654184
	rs1385875	T	0.123722	0.0118693	1.93E-25	-0.00024541	0.000246545	0.32	0.007638876	20.31609478
	rs1960217	T	0.248607	0.0122568	1.80E-91	-0.000478399	0.0002608	0.0669993	0.028321337	76.92572781
	rs3212870	T	0.200682	0.0196896	2.15E-24	-0.000186288	0.000400672	0.64	0.007305959	19.42416362
	rs518418	C	0.196566	0.0127428	1.10E-53	-0.00103117	0.000266247	0.000109999	0.016578534	44.49251609
	rs606555	C	0.254464	0.0172095	1.79E-49	-0.000958492	0.00036561	0.00879995	0.015253217	40.88061258
	rs636800	G	0.127656	0.0131503	2.80E-22	-0.00049798	0.00027389	0.0690001	0.006631982	17.62031674
	rs72932461	A	0.115215	0.0122967	7.28E-21	-0.000671309	0.000258736	0.00949992	0.006181161	16.4150929
	rs75915166	A	-0.206789	0.0248708	9.21E-17	-0.000655133	0.000541594	0.23	0.004873837	12.92627553
**FNTB**	rs112306494	C	0.423809	0.0140751	3.54E-199	0.000482712	0.000308516	0.12	0.060355968	58.87046971
	rs112410305	T	0.92369	0.0314	3.35E-190	0.000905077	0.000703799	0.2	0.0577659	56.18926724
	rs1957949	T	-0.271181	0.0121911	1.28E-109	0.000153005	0.000250357	0.54	0.033868106	32.12884664
	rs4902366	G	0.821412	0.0218277	1.00E-200	5.22E-05	0.000542506	0.92	0.091181059	91.9534231
	rs56328485	T	0.43856	0.0185452	1.24E-123	0.000382863	0.000392885	0.33	0.03811016	36.31249332
	rs58539554	T	1.20086	0.0216613	1.00E-200	0.000501284	0.000551255	0.36	0.178805443	199.5608343
	rs7145602	T	0.361183	0.0158659	1.02E-114	9.89E-05	0.00035905	0.780001	0.035415012	33.65019139
	rs72625654	T	0.687041	0.0221609	1.00E-200	0.000696498	0.000491116	0.16	0.06375293	62.40944473
	rs945015	G	-0.409415	0.0157478	5.19E-149	-0.000324202	0.00033768	0.34	0.045697752	43.88837751
HSPA5	rs2416955	C	-0.110572	0.0176384	3.64E-10	0.000166648	0.000346788	0.630001	0.002776389	22.04952472
	rs4838254	G	0.102549	0.0123654	1.10E-16	-0.000188776	0.000250528	0.450001	0.004849002	38.59000424
	rs55812349	A	0.230373	0.0240285	9.03E-22	-0.000873279	0.000514177	0.089	0.006470089	51.57518374
	rs8759	T	0.623614	0.0418637	3.49E-50	-0.00106187	0.000827712	0.2	0.015477549	124.5053557
**ITGAL**	rs112929095	A	0.276298	0.0142665	1.47E-83	0.000714935	0.000305131	0.0189998	0.025885195	52.59469563
	rs113303075	A	0.243212	0.0276199	1.30E-18	0.00155468	0.000541257	0.00409996	0.005463444	10.87292591
	rs1133238	A	-0.235187	0.0227001	3.75E-25	0.000106472	0.000578329	0.85	0.00754742	15.05183314
	rs11863188	T	-0.173155	0.0168896	1.16E-24	4.30E-05	0.000393682	0.91	0.007391436	14.73843721
	rs12919022	C	-0.201112	0.0226846	7.60E-19	0.000365831	0.000528933	0.49	0.005537617	11.02136055
	rs17790434	T	0.162546	0.0148786	8.77E-28	0.000138919	0.000291865	0.630001	0.008384777	16.73589653
	rs3087439	T	0.242976	0.0225745	5.14E-27	0.000533592	0.000463905	0.25	0.008140656	16.24463637
	rs35493324	T	-0.298999	0.0154421	1.60E-83	-8.44E-05	0.000330489	0.8	0.025873708	52.5707366
	rs56391383	A	0.140439	0.0123987	9.67E-30	0.000225983	0.000251177	0.37	0.009007655	17.99045209
	rs9746755	G	0.111176	0.0118757	7.84E-21	1.53E-05	0.000247323	0.95	0.006170744	12.28927897
**NEU1**	rs1270942	G	-0.162245	0.0194613	7.62E-17	0.000882009	0.000365982	0.016	0.005467747	13.06279096
	rs3130490	T	-0.171438	0.0196036	2.22E-18	0.000840074	0.000365015	0.021	0.006001875	14.3465609
	rs542418	G	-0.0940021	0.0127811	1.91E-13	-3.83E-05	0.000258852	0.88	0.005578681	13.32930632
	rs592229	T	0.0689131	0.0120188	9.82E-09	-0.000154746	0.00024574	0.53	0.005443198	13.00382063
**PTGS1**	rs10306194	A	0.147988	0.0186399	2.03E-15	0.000237751	0.000364117	0.51	0.00444579	14.14403273
	rs10818695	G	0.106517	0.0120226	8.03E-19	-2.86E-05	0.000248135	0.91	0.005530318	17.61358497
	rs146145158	T	-0.307432	0.0345698	5.94E-19	-0.00128369	0.000849935	0.13	0.005571838	17.74656337
	rs2487474	C	-0.105011	0.0134567	6.01E-15	-0.000126687	0.000281764	0.649999	0.004295782	13.66473086
	rs35918962	T	0.184086	0.0179987	1.49E-24	0.000803996	0.000374808	0.032	0.007356469	23.47282182
	rs5788	A	-0.189717	0.0170969	1.30E-28	-0.000528316	0.000378595	0.16	0.008648232	27.63050028
	rs62575596	A	-0.232359	0.018252	3.99E-37	-0.000587633	0.00038335	0.13	0.011351668	36.3669635
	rs72769724	A	-0.302697	0.0431672	2.35E-12	0.000848467	0.000934352	0.36	0.003471497	11.03357459

MR analysis was performed using IVW, MR Egger and weighted median methods. IVW analysis of target genes in relation to ovarian cancer showed that HMGCR, PLA2G4A, ITGAL, PTGS1, FNTA, FNTB, HSPA5, NEU1, CCND1, CASP1, CASP3 were significantly associated in ovarian cancer had significant correlation. Among them, FNTA, HSPA5, NEU1, CCND1, CASP1, CASP3 had a negative causal association with ovarian cancer development, and HMGCR, PLA2G4A, ITGAL, PTGS1, FNTB had a positive causal association with ovarian cancer development.

**Figure 2:**
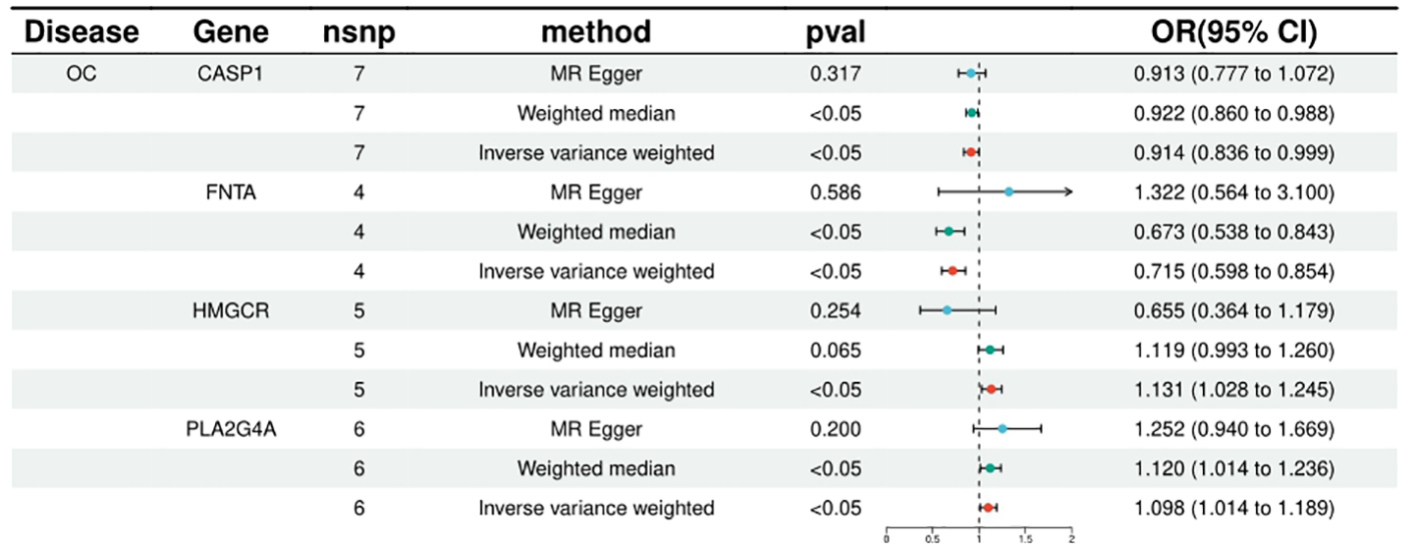
Summary of the univariable MR analysis results for the association between drug-related targets and the risk of Ovarian cancer (ieu-a-1120) using the IVW, MR Egger, and Weighted median methods.

FNTA, HSPA5, NEU1, CCND1, CASP1, CASP3 were included in the analyses of 4, 4, 4, 9, 7 and 11 SNPs respectively, with statistically significant IVW results for FNTA (ORIVW = 0.714571216, 95% CI = 0.597682908 - 0.854319265; PIVW = 0.000226306), HSPA5 (ORIVW = 0.997892376, 95% CI = 0.995974116- 0.999814331; PIVW = 0.031622971), NEU1 (ORIVW = 0.996386436, 95% CI = 0.993805213- 0.998974362; PIVW = 0.006230918), CCND1 (ORIVW = 0.996798602, 95% CI = 0.995203219- 0.998396544; PIVW = 8.72E-05), CASP1 ORIVW = 0.914123044, 95% CI = 0.836059318 - 0.999475662; PIVW= 0.048664899), CASP3 (ORIVW = 0.999417702, 95% CI = 0.998700179- 0.999846044; PIVW = 0.012906474), the statistical results showed a negative correlation between these targets and ovarian cancer risk. (**Figure 2**; **Supplementary Table 2**).

**Figure 3:**
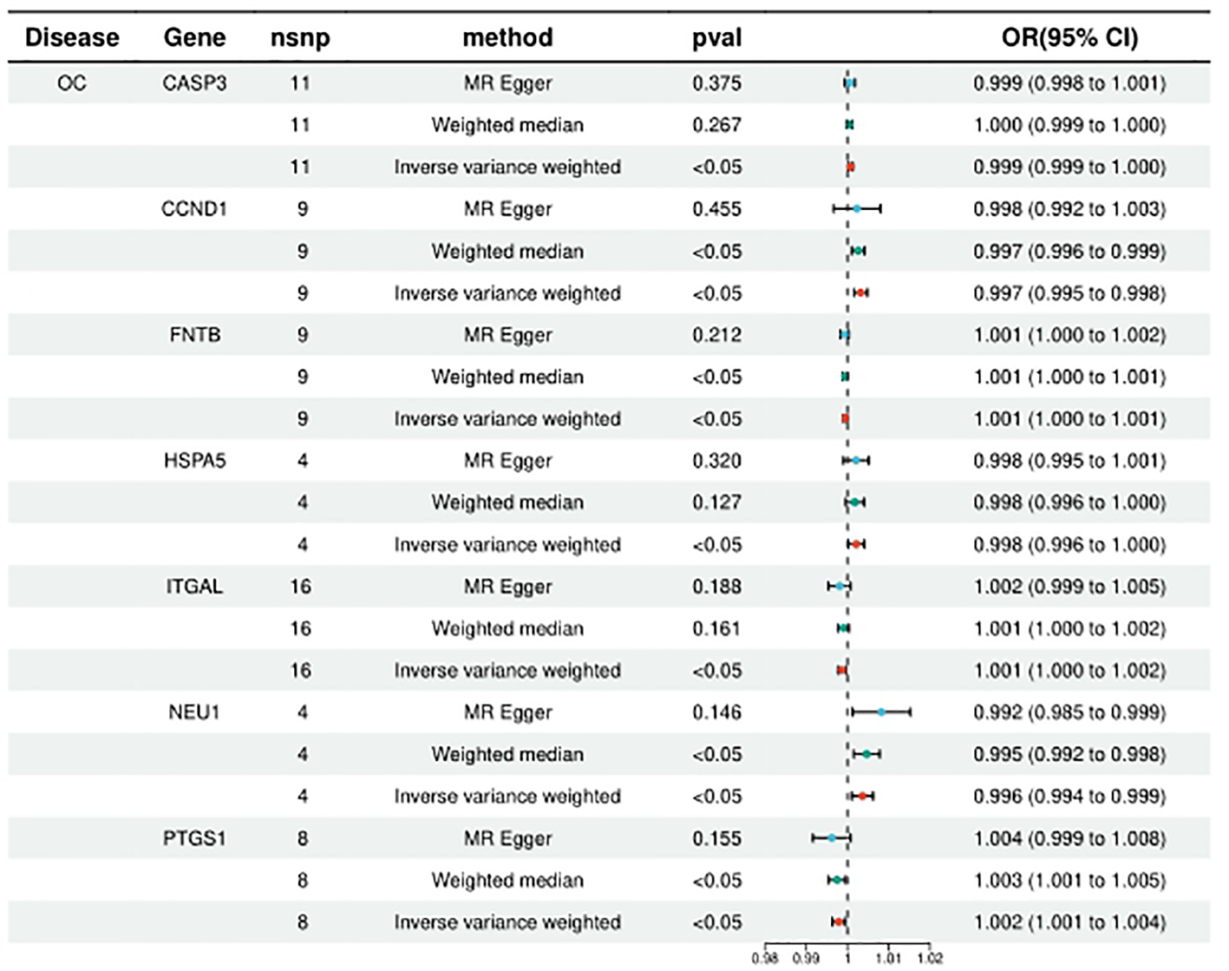
Summary of the univariable MR analysis results for the association between drug-related targets and the risk of Ovarian cancer (ieu-b-4963) using the IVW, MR Egger, and Weighted median methods.

HMGCR, PLA2G4A, ITGAL, PTGS1, and FNTB were included in the analysis of 5, 6, 10, 8, and 9, respectively, and the statistical results of IVW were, respectively, HMGCR (ORIVW = 1.131314709, 95% CI = 1.027776107 - 1.027776107; PIVW = 0.011753459), PLA2G4A (ORIVW = 1.097906571, 95% CI = 1.013595152- 1.189231062; PIVW = 0.021949277), ITGAL (ORIVW = 1.001353198, 95% CI = 1.000217005 - 1.002490683; PIVW = 0.000579236), PTGS1 (ORIVW = 1.002096738, 95% CI = 1.000537301-1.003658604; PIVW = 0.008388408), FNTB (ORIVW = 1.000560816, 95% CI = 1.000092211 - 1.001029641; PIVW = 0.018986672). It is suggested that increased expression of these targets may promote ovarian carcinogenesis, while targeted inhibition of these targets may prevent or reduce ovarian carcinogenesis. (**Figure 3**; **Supplementary Table 2**).

### Results of sensitivity analyses

The results of MR-PRESSO analysis showed that there were no outliers for all SNPs included in this study. Heterogeneity test was conducted using the IVW method and MR-Egger regression, when the test P < 0.05, heterogeneity exists among SNPs; when the P value > 0.05, heterogeneity does not exist among SNPs (**Supplementary Figure 1**). MR-Egger regression was used to test for polytropy, when the test P < 0.05, it means that there is polytropy; when the P value > 0.05, it means that there is no polytropy. (**Supplementary Figure 2**) Leave-one-out analysis was used to remove one SNP at a time, and the remaining SNPs were subjected to IVW analysis. (**Supplementary Figure 3**,**Supplementary Table 3**).

## Discussion

Currently, the role of drug repositioning in the treatment of ovarian cancer is gradually attracting attention, but there is a lack of large-scale, multicentre, prospective studies, and the retrospective studies are affected by a variety of confounding factors, leading to huge discrepancies in the results of current studies. Drug-targeted Mendelian randomisation studies are based on the downstream products of target proteins (biomarker), SNPs (pQTL or eQTL) with significant effects on biomarker near the gene encoding the target protein are used as instrumental variables, and Mendelian randomisation analyses are performed with disease as the endpoint to validate the effects of the target proteins on the disease under study.

By analysis, we found that HMGCR, PLA2G4A, ITGAL, NR3C1, PTGS1, FNTA, FNTB, HSPA5, NEU1, CCND1, CASP1, CASP3 were significantly correlated with ovarian carcinogenesis. Among them, HMGCR is an important target of various statins, ITGAL is mainly a target of fat-soluble statins (e.g., lovastatin, pitavastatin), and FNTA and FNTB are targets of the MVA pathway-related enzyme inhibitor lonafarnib (22). PLA2G4A is a target of the anti-malarial drug adiponectin, PTGS1, HSPA5, NEU1, CCND1, CASP1, CASP3 is an important target of aspirin.

The main target of action of statins is HMGCR, but they can also act on other targets, such as HDAC2, DPP4, ITGAL and so on. Among these, ITGAL encodes integrin α, which is the target of fat-soluble statins. Previously, it was thought that only fat-soluble statins have an anti-ovarian cancer effect and can prolong the generation time of patients (23). It has been suggested that this may be due to the fact that lipid-soluble drugs can cross the cell membrane more easily and thus exert anti-tumour effects (24). The results of the present study suggest that lipid-soluble statins may exert anti-tumour effects not only by interfering with HMGCR, but also, possibly, by inhibiting ITGAL.

As statins are key enzymes in the MVA pathway, it has long been questioned whether similar anti-tumour effects could be achieved by inhibiting other enzymes in the pathway (25), or whether better therapeutic effects could be achieved by combining drugs (26). It has also been suggested that the anti-tumour effects of statins may not be related to lipid lowering but to their intermediate metabolites such as FPP and GGPP, which provide isoprenyl groups to isoprenylate a variety of small GTPase-binding proteins such as Ras, MEK, PI3K, and anchor them to the cell membrane. This is how it works, and some studies suggest that zoledronic acid and lonafarnib may reduce the production of FPP and GGPP, thereby exerting an anti-ovarian cancer effect. However, it has also been suggested that the anti-tumour effect of statins can only be reversed by the addition of GGPP (24). In this study, we also found that the main targets of zoledronic acid, GGPS1 and FDPS, were not associated with the development of ovarian cancer, but the targets of action of lonafarnib, the two subunits of farnesyltransferase, FNTA and FNTB, were both associated with the development of ovarian cancer, which seems to support this view. However, it is difficult to explain why FNTA is negatively associated with ovarian cancer prognosis, while FNTB is positively associated with ovarian carcinogenesis.

The mechanism by which aspirin improves the prognosis of ovarian cancer is usually thought to be related to COX inhibition, with low-dose aspirin primarily inhibiting COX1 and higher doses inhibiting COX2 activity. In the past, the literature has suggested that high doses of aspirin reduced mortality in patients of ovarian cancer (19). More recent literature suggests that the source of the statistics may amplify this dose-dependent difference (27). Our study shows that PTGS1 (COX1) is significantly positively correlated with ovarian cancer prognosis, while PTGS2 is not significantly correlated with ovarian cancer prognosis. In addition aspirin may inhibit several targets such as HSPA5, NEU1, CCND1, CASP1, CASP3, which are negatively correlated with ovarian cancer prognosis. Therefore, whether simply acting on PTGS1 targets can obtain better anti-ovarian cancer effects will be a question we need to verify in the future.

Originally used in antimalarial therapy, quinacrine has now been shown to have anti-tumour effects (28, 29), particularly in inducing apoptosis in P53-deficient or mutated tumour cells (30). PLA2G4A encodes phospholipase A2, and quinacrine may inhibit ovarian cancer by inhibiting PLA2G4A which in turn reduces arachidonic acid metabolites and thus inhibits ovarian cancer development.

### Limitation of the study

Currently, drug target Mendelian randomisation analysis usually selects single-target drugs, such as PCSK9 inhibitors, while statins are considered to be single-target drugs for HMGCR (20), but statins are not actually single-target drugs, and their targets of action are not limited to HMGCR. there are also drugs for which it is difficult to specify which target is the main target of their action. Therefore, in this study, all known targets were screened for target selection. However, whether this affects the results is also a problem that needs to be addressed in the current Mendelian randomization analysis of drug targeting.

In addition, when selecting genetic association data for ovarian cancer, we selected two datasets, but found that the conclusions drawn from the two databases were not consistent. The number of ovarian cancer patients and SNP loci found in ieu-a-1120 data far exceeds that in ieu-b-4963. It suggests that the results of drug-targeted Mendelian randomisation analysis are affected by multiple factors (sequencing technology, sequencing depth and number of cases).

Third, the genetic information related to ovarian cancer is mainly from European populations, but the results of drug repositioning may be affected by regional and racial differences as reported in the relevant literature. Further studies are needed to clarify the differences between different races.

Therefore, the results of this study are only for reference, and we hope to provide ideas for the selection of therapeutic targets for ovarian cancer in the future.

## Conclusion

This study provides MR evidence for the potential value of conventional drugs in preventing ovarian cancer. Genetic links between drug targets and ovarian cancer may accelerate drug development prioritisation and reduce the failure rate of clinical trials and provide new ideas for future drug development.

## Data availability statement

The original contributions presented in the study are included in the article/**Supplementary Material**. Further inquiries can be directed to the corresponding authors.

## Ethics statement

Ethical approval was not required for the study involving humans in accordance with the local legislation and institutional requirements. Written informed consent to participate in this study was not required from the participants or the participants’ legal guardians/next of kin in accordance with the national legislation and the institutional requirements.

## Author contributions

LZ: Writing – original draft, Data curation, Software. HZ: Writing – original draft, Conceptualization, Investigation, Software. XZ: Data curation, Software, Writing – original draft. RC: Data curation, Investigation, Writing – original draft. LX: Supervision, Validation, Writing – review & editing.

## Supplementary material

Supplementary Figure 1 IVW and MR-Egger regression heterogeneity test chart.

Supplementary Figure 2 Scatter plot of Mendelian randomization analysis.

Supplementary Figure 3 Analysis diagram of leave-one-out method.
