# Supplementary figures and images for: Drug repositioning and ovarian cancer, a study based on Mendelian randomisation analysis
Source: Front Oncol. 2024 Apr 8;14:1376515. doi: 10.3389/fonc.2024.1376515 (PMC11033362; doi:10.3389/fonc.2024.1376515)

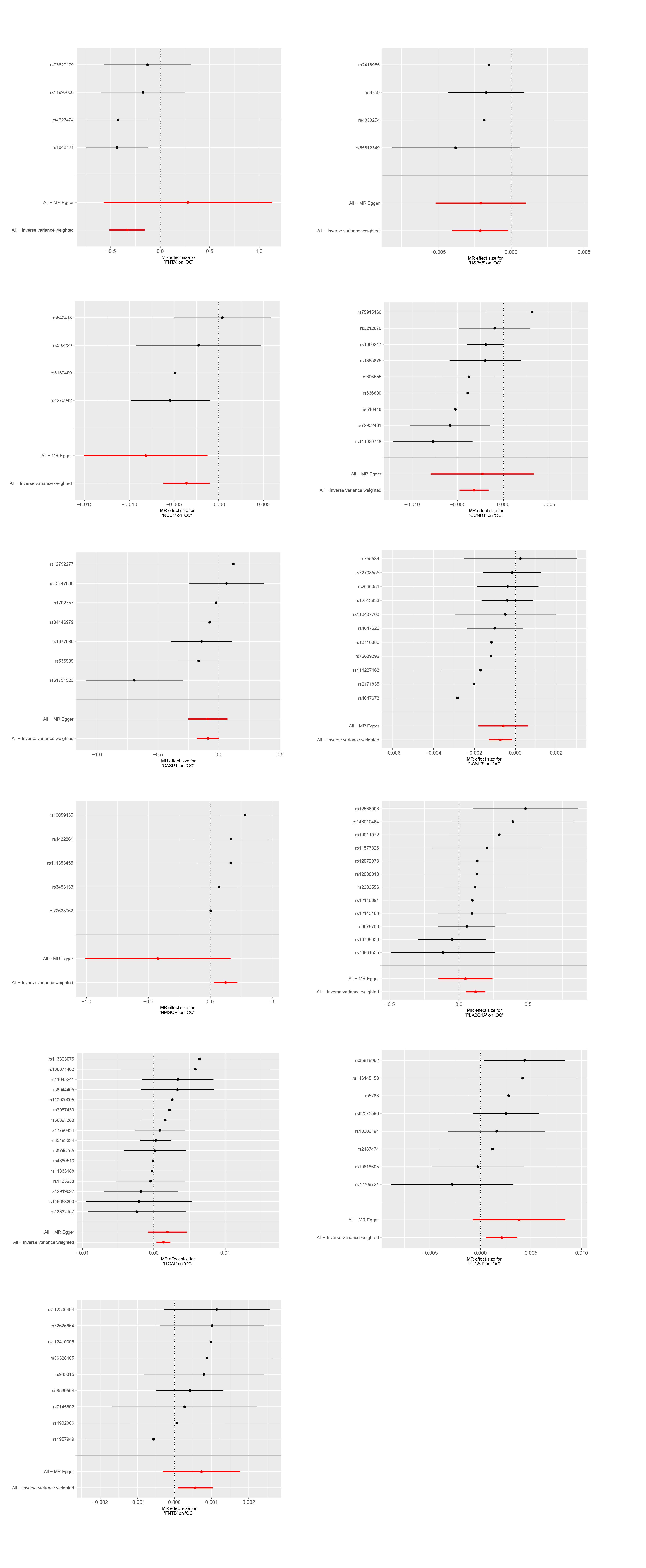

Supplement: Supplementary Figure 1 — IVW and MR-Egger regression heterogeneity test chart. [file Image_1.jpeg]

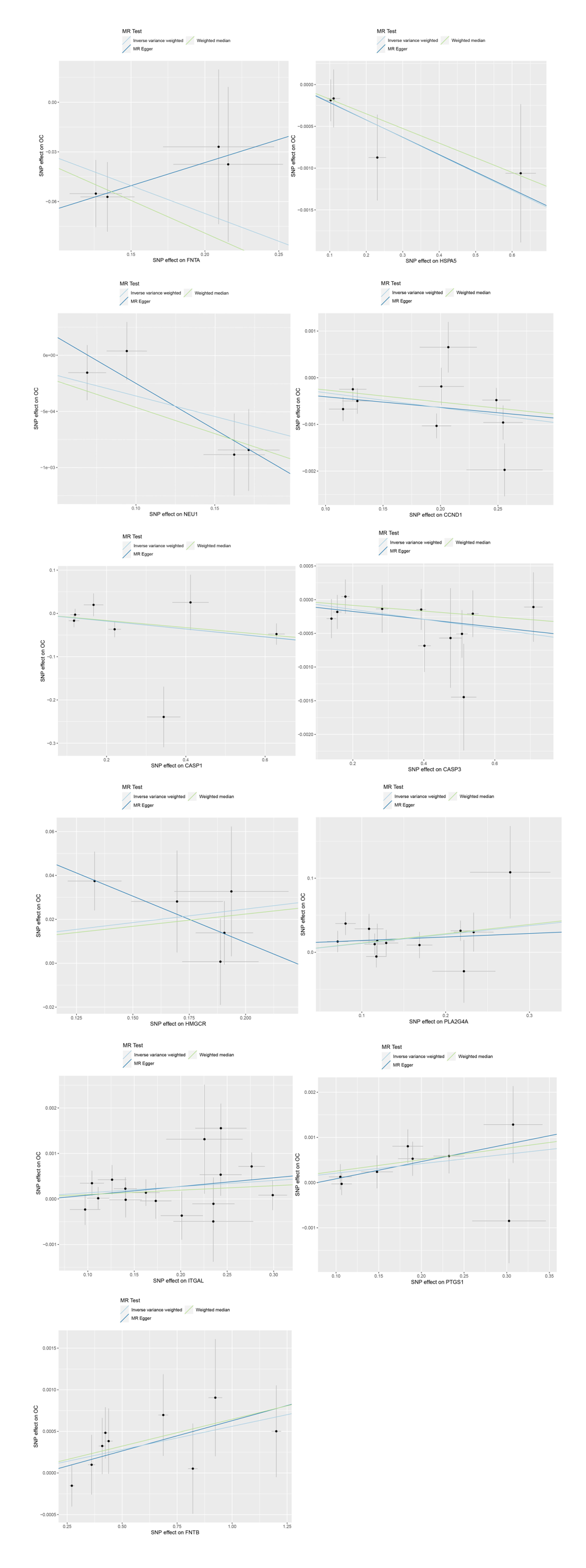

Supplement: Supplementary Figure 2 — Scatter plot of Mendelian randomization analysis. [file Image_2.jpeg]

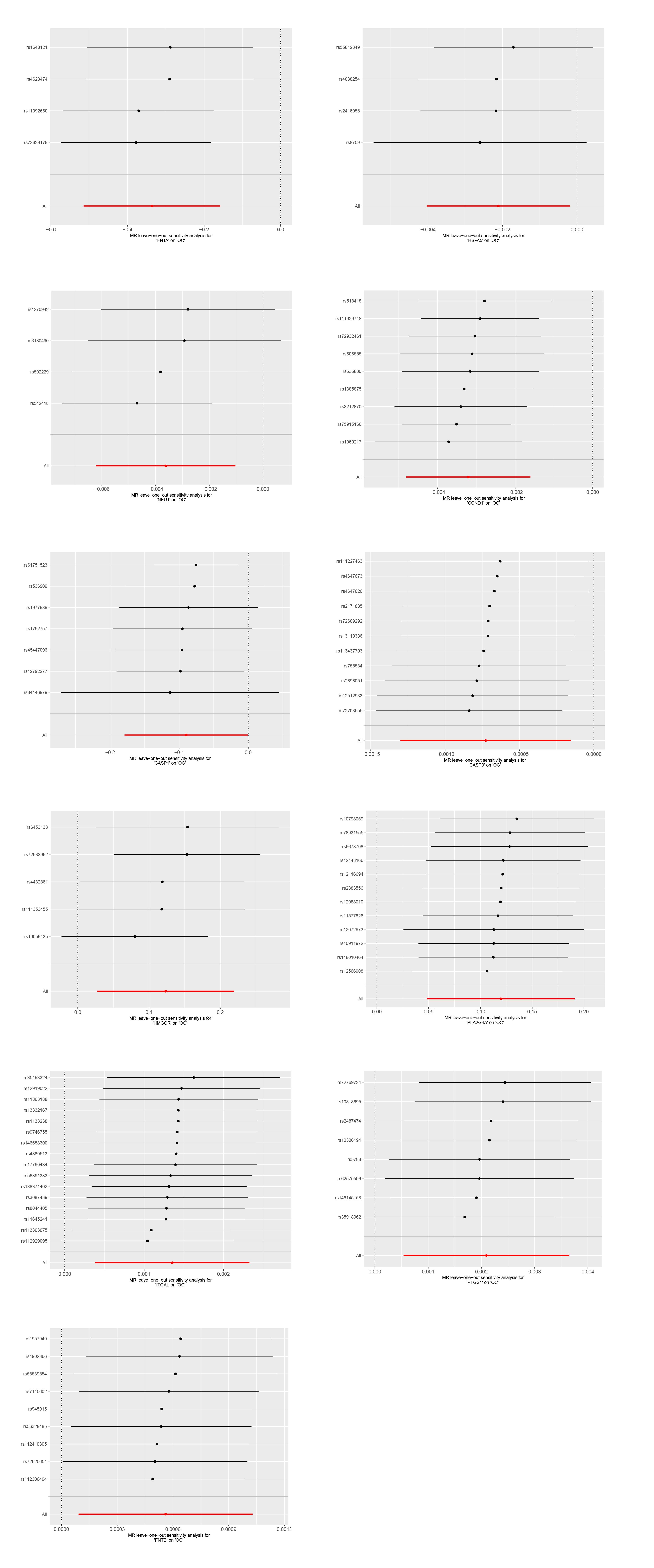

Supplement: Supplementary Figure 3 — Analysis diagram of leave-one-out method. [file Image_3.jpeg]
