# Supplementary material for: Drug repositioning and ovarian cancer, a study based on Mendelian randomisation analysis
Source: Front Oncol. 2024 Apr 8;14:1376515. doi: 10.3389/fonc.2024.1376515 (PMC11033362; doi:10.3389/fonc.2024.1376515)
Supplement: Supplementary file 4 [file Table_1.docx]

Supplementary Table1 Drug repositioning in ovarian cancer and targets

| **Drug** | **drugID** | **Gene** | **action** |
| --- | --- | --- | --- |
| atorvastatin | DB01076 | HMGCR | inhibitor |
|  |  | DPP4 | inhibitor |
|  |  | AHR | agonist |
|  |  | HDAC2 | inhibitor |
|  |  | NR1I3 | ligand |
| lovastatin | DB00227 | HMGCR | inhibitor |
|  |  | ITGAL | inhibitor |
|  |  | HDAC2 | inhibitor |
| Mebendazole | DB00643 | TUBA1A | inhibitor |
|  |  | TUBB4B | inhibitor |
| Apomorphine | DB00714 | DRD4 | agonist |
|  |  | DRD2 | agonist |
|  |  | DRD3 | agonist |
|  |  | DRD5 | agonist |
|  |  | DRD1 | agonist |
|  |  | P18825 | agonist |
|  |  | ADRA2B | agonist |
|  |  | HTR1A | agonist |
|  |  | HTR2A | agonist |
|  |  | HTR2B | agonist |
|  |  | HTR2C | agonist |
|  |  | ADRA2A | agonist |
|  |  | HTR1D | agonist |
|  |  | HTR1B | agonist |
| Clofazimine | DB00845 | PPARG | Modulator |
|  |  | KCNA3 | Antagonist |
| Disulfiram | DB00822 | DBH | inhibitor |
|  |  | ALDH2 | inhibitor |
| Digitoxin | DB01396 | ATP1A1 | inhibitor |
| CDB-2914 | DB08867 | PGR | Modulator |
|  |  | NR3C1 | antagonist |
|  |  | AR | unknown |
| Quinacrine | DB01103 | PLA2G6 | inhibitor |
|  |  | PLA2G4A | inhibitor |
|  |  | PLCL1 | inhibitor |
| Itraconazole | DB01167 | ERG11 | inhibitor |
|  |  | CYP51A1 | inhibitor |
| Hydroxychloroquine | DB01611 | TLR7 | antagonist |
|  |  | TLR9 | antagonist |
|  |  | ACE2 | Modulator |
| Metformin | DB00331 | ETFDH | inhibitor |
|  |  | PRKAB1 | inducer/activator |
|  |  | GPD1 | inhibitor |
| anastrozole | DB01217 | CYP19A1 | inhibitor |
| aspirin | DB00945 | PTGS1 | inhibitor |
|  |  | PTGS2 | inhibitor |
|  |  | AKR1C1 | inhibitor |
|  |  | EDNRA | inhibitor |
|  |  | TP53 | inducer |
|  |  | HSPA5 | inhibitor |
|  |  | RPS6KA3 | inhibitor |
|  |  | NFKB1A | inhibitor |
|  |  | TNFA1P6 | inhibitor/downregulator |
|  |  | CASP1 | inhibitor/downregulator |
|  |  | CASP3 | inhibitor/downregulator |
|  |  | IKBKB | unknown |
|  |  | ERK | unknown |
|  |  | CCND1 | downregulator |
|  |  | MYC | downregulator |
|  |  | PCNA | downregulator |
|  |  | NEU1 | inhibitor |
| vitamin D | DB11094 | VDR | unknown |
|  |  | GC | unknown |
| zoledronic acid | DB00399 | GGPS1 | inhibitor |
|  |  | FDPS | inhibitor |
| CEP-1347 | DB05403 | MAPK12 | unknown |
| Captopril | DB01197 | ACE | inhibitor |
|  |  | MMP2 | inhibitor |
|  |  | MMP9 | inhibitor |
|  |  | LTA4H | inhibitor |
|  |  | BDKRB1 | unknown |
| Enalaprilat | DB09477 | ACE | inhibitor |
|  |  | BDKRB1 | unknown |
| manidipine | DB09238 | CACNA1G | inhibitor |
|  |  | CACNA1H | inhibitor |
|  |  | CACNA1I | inhibitor |
| lacidipine | DB09236 |  |  |
| benidipine | DB09231 | CACNA1B | antagonist |
| lomerizine | DB14065 | ABCB1 | inhibitor |
| amiodarone | DB01118 | CACNA1I | inhibitor |
|  |  | PPARG | agonist |
|  |  | PPARA | agonist |
|  |  | PPARGC1B | agonist |
| dronedarone | DB04855 | ADA1A | antagonist |
|  |  | ADA1B | antagonist |
|  |  | ADA1D | antagonist |
|  |  | ADRA2A | antagonist |
|  |  | ADRA2B | antagonist |
|  |  | ADRA2C | antagonist |
|  |  | KCNH2 | inhibitor |
|  |  | CACNA1C | inhibitor |
|  |  | CACNA1D | inhibitor |
|  |  | CACNA1F | inhibitor |
|  |  | CACNA1S | inhibitor |
|  |  | CACNB1 | inhibitor |
|  |  | CACNB2 | inhibitor |
|  |  | CACNB3 | inhibitor |
|  |  | CACNB4 | inhibitor |
|  |  | KCNK2 | inhibitor |
|  |  | SCN5A | inhibitor |
|  |  | KCNJ3 | inhibitor |
|  |  | KCNQ1 | inhibitor |
|  |  | SLC8A1 | inhibitor |
|  |  | KCND3 | inhibitor |
|  |  | ADRB1 | antagonist |
|  |  | THRA | inhibitor |
| OLANZAPINE | DB00334 | HRT2A | antagonist |
|  |  | DRD2 | antagonist |
|  |  | DRD1 | antagonist |
|  |  | DRD5 | antagonist |
|  |  | DRD3 | antagonist |
|  |  | DRD4 | antagonist |
|  |  | HTR2C | antagonist |
|  |  | HTR2A | antagonist |
|  |  | HTR6 | antagonist |
|  |  | HRH1 | antagonist |
|  |  | ADRA1A | antagonist |
|  |  | ADA1B | antagonist |
|  |  | CHRM1 | antagonist |
|  |  | CHRM2 | antagonist |
|  |  | CHRM3 | antagonist |
|  |  | CHRM4 | antagonist |
| Ceritinib | DB09063 | ALK | Ceritinib |
| Ritonavir | DB00503 | NRI12 | activator |
| Ropinirole | DB00268 | DRD3 | agonist |
|  |  | DRD2 | agonist |
|  |  | DRD4 | agonist |
| pitavastatin | DB08850 | HMGCR | inhibitor |
|  |  | ITGAL | inhibitor |
| Lonafarnib | DB06448 | FNTA | inhibitor |
|  |  | FNTB | inhibitor |
