# Supplementary material for: Drug repositioning and ovarian cancer, a study based on Mendelian randomisation analysis
Source: Front Oncol. 2024 Apr 8;14:1376515. doi: 10.3389/fonc.2024.1376515 (PMC11033362; doi:10.3389/fonc.2024.1376515)
Supplement: Supplementary file 5 [file Table_2.docx]

| **Target** | **Method** | **Ovarian cancer**  **SNP β SE P** | | | |
| --- | --- | --- | --- | --- | --- |
| CASP1 | IVW  MR Egger  WM | 7  7  7 | -0.089790095  -0.091117401  -0.081679492 | 0.045543683  0.081994661  0.035446045 | 0.048664899  0.317015322  0.021203957 |
| FNTA | IVW  MR Egger  WM | 4  4  4 | -0.336072615  0.279349998  -0.395524798 | 0.091133829  0.434757645  0.114431445 | 0.000226306  0.586347764  0.000547372 |
| HMGCR | IVW  MR Egger  WM | 5  5  5 | 0.123380416  -0.422398388  0.111993353 | 0.048970952  0.299736702  0.060689218 | 0.011753459  0.253537673  0.253537673 |
| PLA2G4A | IVW  MR Egger  WM | 6  6  6 | 0.119788486  0.04725146  0.125170325 | 0.036393897  0.099715979  0.050928373 | 0.000996744  0.645775589  0.013980193 |
| CASP3 | IVW  MR Egger  WM | 11  11  11 | -0.000727317  -0.000582468  -0.000422957 | 0.000292525  0.000623547  0.000380887 | 0.012906474  0.374625243  0.266804171 |
| CCND1 | IVW  MR Egger  WM | 9  9  9 | -0.003206533  -0.002290272  -0.002607366 | 0.00081724  0.002893921  0.00079554 | 8.72E-05  0.45468177  0.001047389 |
| FNTB | IVW  MR Egger  WM | 9  9  9 | 0.000560659  0.000727313  0.000645835 | 0.000239006  0.000529165  0.00029899 | 0.018986672  0.211686656  0.030768309 |
| HSPA5 | IVW  MR Egger  WM | 4  4  4 | -0.002109848  -0.002072965  -0.001751919 | 0.000981715  0.001580393  0.001084341 | 0.031622971  0.319972673  0.106169245 |
| ITGAL | IVW  MR Egger  WM | 10  10  10 | 0.001354396  0.001910022  0.000969277 | 0.000496245  0.001380161  0.000680849 | 0.000996744  0.188052128  0.006347071 |
| NEU1 | IVW  MR Egger  WM | 4  4  4 | -0.003620109  -0.008173861  -0.004636972 | 0.001323441  0.003527085  0.001680243 | 0.006230918  0.146389084  0.0057854 |
| PTGS1 | IVW  MR Egger  WM | 8  8  8 | 0.002094542  0.003810827  0.002535473 | 0.000794584  0.002343319  0.001037337 | 0.008388408  0.155020826  0.014516832 |

Supplementary Table2 Results of univariate Mendelian randomization studies
