# Supplementary material for: Drug repositioning and ovarian cancer, a study based on Mendelian randomisation analysis
Source: Front Oncol. 2024 Apr 8;14:1376515. doi: 10.3389/fonc.2024.1376515 (PMC11033362; doi:10.3389/fonc.2024.1376515)
Supplement: Supplementary file 6 [file Table_3.docx]

Supplementary Table3 Sensitivity analysis of MR Studies

| **Target** | **Outcome** | **Heterogeneity test** | | **MR Egger** | | **MR-PRESSO test** | |
| --- | --- | --- | --- | --- | --- | --- | --- |
|  |  | **MR Egger IVW** | | **Intercept P** | | **Outliers P** | |
| CASP1 | OC | P=0.023044867 | P=0.042452723 | 0.000422192 | 0.984515036 | None | 0.171 |
| FNTA | OC | P=0.9722705 | P=0.541439155 | -0.092317288 | 0.28466436 | None | 0.624 |
| HMGCR | OC | P=0.79711187 | P=0.352767746 | 0.093923143 | 0.162505553 | None | 0.383 |
| PLA2G4A | OC | P=0.915404651 | P=0.871462219 | -0.022330225 | 0.403163552 | None | 0.854 |
| CASP3 | OC | P=0.823959406 | P=0.878372335 | -5.84E-05 | 0.798441907 | None | 0.904 |
| CCND1 | OC | P=0.018277804 | P=0.028765341 | -0.000177586 | 0.749714091 | None | 0.057 |
| FNTB | OC | P=0.819885728 | P=0.877482374 | -9.90E-05 | 0.73448332 | None | 0.883 |
| HSPA5 | OC | P=0.701385944 | P=0.870783171 | -8.83E-06 | 0.978946762 | None | 0.86 |
| ITGAL | OC | P=0.269595806 | P=0.31935718 | -0.000225034 | 0.559212311 | None | 0.354 |
| NEU1 | OC | P=0.50002345 | P=0.347930681 | 0.000566849 | 0.300916774 | None | 0.439 |
| PTGS1 | OC | P=0.532158937 | P=0.575490773 | -0.000296885 | 0.465835478 | None | 0.66 |
